# A diluent containing coconut water, fructose, and chicken egg yolk increases rooster sperm quality at 5°C

**DOI:** 10.14202/vetworld.2019.1116-1120

**Published:** 2019-07-25

**Authors:** Siti Eliana Rochmi, Miyayu Soneta Sofyan

**Affiliations:** 1 Department of Health Science, Faculty of Vocational Studies, Universitas Airlangga, Surabaya, Indonesia; 2 Department of Postgraduate Veterinary Sciences, Faculty of Veterinary Medicine, Universitas Airlangga, Surabaya, Indonesia

**Keywords:** chicken, coconut water, diluent, egg yolk, sperm quality

## Abstract

**Aim:**

The present study was conducted to evaluate the quality of rooster sperm at 5°C after treatment with a diluent containing coconut water, fructose, and chicken egg yolk and stored the semen sample at 5°C.

**Materials and Methods:**

Ten semen samples from 10 healthy roosters were subjected to four different treatments. For the treatments, 0.2 ml fresh semen with a sperm concentration of 5.2×10^9^ cell/ml was mixed with T0 (no diluent), T1 (0.34 ml coconut water and 6 µl fructose), T2 (0.274 ml coconut water, 0.12 ml egg yolk, and 6 µl fructose), and T3 (0.34 ml egg yolk and 6 µl fructose) solutions. Each treated solution was stored at 5°C and evaluated both macroscopically and microscopically. Macroscopically, semen volume, pH, and sperm concentration were evaluated. The microscopic sperm characteristics examined included total motility (i.e., rapid, medium, or slow), progressive and non-progressive motility, viability, and spermatozoa abnormalities noted at different storage times. The results showed that spermatozoa motility was under 40%.

**Results:**

The results indicated that sperm viability significantly affected (p<0.05). The highest mean value of sperm viability on day 7 of storage was found after treatment with the T2 solution (46.100±0.5677%). Similarly, spermatozoa abnormalities were significantly lower after treatment with the T2 solution (6.680±1.702%).

**Conclusion:**

The addition of a diluent containing coconut water, egg yolk, and fructose helped in the better preservation spermatozoa motility, as well as viability for up to 7 days when the semen samples were stored at 5°C.

## Introduction

Chicken products (such as eggs and meat) are staple Indonesian foods. This livestock possesses high commodity value. However, this market is plagued by some intrinsic weaknesses such as low production, slow growth, nature of incubation, and poor genetic quality [1]. Artificial insemination (AI) is a biotechnological method used to increase chicken productivity and yield. Storage parameters such as temperature, diluent, and energy sources for spermatozoa form the most important parameters for the success of AI [2,3]. In addition, semen quality is affected by various factors such as breed, age, feed, environmental stressors such as temperature and humidity, sperm dose, sperm disposition, and storage time before AI [4,5]. Spermatozoa storage systems (capable of maintaining spermatozoa fertilizing ability *in vitro* for 24 h) are hardly as efficient as the oviductal storage systems (an *in vivo* system that can maintain fertilizing ability for many weeks) [6]. Environmental temperature is an important factor that influences the semen quality and fertility of roosters in tropical countries where birds are primarily raised in open poultry houses. Semen quality in roosters is reported to decrease during summer or upon exposure to high temperatures. Heat exposure may result in nuclear abnormalities, thus leading to declined fertility [5].

Spermatozoa motility is critical for the maintenance of fertility. The vaginal segment of a hen’s oviduct regulates spermatozoa, and only motile spermatozoa will traverse the vagina and enter the hen’s sperm storage tubules [7]. Motility of spermatozoa is a primary determinant of fertility in fowl [8], turkeys [9], Venda chickens [10], and roosters [11,12]. A graded influence was predicted between fertility and spermatozoa motility when fertility was plotted as a function of spermatozoa motility. Semen diluents are added to maintain sperm motility and fertility. Most such diluents provide energy for metabolism. They also provide buffering capacity [13], prevent clumping by thinning out the sperm concentration, and increase the metabolic activity of the spermatozoa while enhancing the motility. From a practical viewpoint, this helps in reducing the number of males required to fertilize females as well as the overall costs [9]. For their effective utilization, diluents must be maintained at the same pH as that of semen, protected of sperm from cold shock, and contain nutrients for spermatozoa [14]. Some of the diluents employed are egg yolk, milk, and coconut water.

The majority of diluents used are salt solutions. As these solutions provide an osmotic pressure of 300-400 mOsm and a pH of 7.0-7.4, they can be conveniently used for the short-term storage of spermatozoa [15]. An ideal diluent should also contain various energy substrates; therefore, those used for poultry semen are enriched with carbohydrates (glucose or fructose) and other components that are likely to provide energy in the form of citrate, glutamate, or acetate [9]. Coconut water is a natural isotonic liquid containing glucose, minerals, vitamins, and proteins and is widely used as a substitute for lost body fluids and prevention of poisoning, particularly mineral poisoning [16]. Fructose is added to the diluent because the sperm plasma contains a variety of specific organic compounds, one of which is fructose. Egg yolk is a good diluent because it is affordable and accessible, has good energy source, and contains nearly the same physical and biochemical elements as sperm [17].

This study aimed to evaluate the effects of a diluent containing coconut water, fructose, and chicken egg yolk on the sperm quality of a local rooster when the semen samples were stored at 5°C.

## Materials and Methods

### Ethical approval

Ethical approval is not required for this type of study. However, semen samples were collected as per standard collection procedure.

### Animals

In this study, semen from 10 healthy roosters (n=10) was collected. The following criteria had to be met for collection: The roosters were 1 year old and possessed shiny fur, glowing eyes, high libido, and display agile movement. The animals were housed in conventional individual cages under 14 h of daily illumination and fed with a standard commercial food at a rate of 155 g/day/animal.

### Sperm collection

Semen was collected in a glass funnel by the dorso-abdominal massage method [5,18]. Semen collection was conducted by stimulating the copulatory organ until protrusion; this was done by massaging the abdomen and the back above the testes. The semen used in this research was collected twice a week for 5 weeks, and the experiment was repeated 10 times. To minimize animal stress, the collection was carried out by the same operator and under the same conditions.

### Sperm treatment

The pooled semen was transferred to a water bath (37°C) and evaluated via macroscopic and microscopic examination. The parameters examined included amount ejaculate volume, color, consistency, smell, pH, sperm concentration, motility of mass, sperm motility, viability, and abnormalities. After these evaluations, the semen samples were subjected to different treatments. Each treatment consisted of 0.2 ml fresh semen (sperm concentration 5.2×10^9^ cell/ml) mixed with either of the following: T0: no diluent; T1: 0.34 ml coconut water and 6 µl fructose; T2: 0.27 ml coconut water, 0.12 ml egg yolk, and 6 µl fructose; T3: 0.34 ml egg yolk and 6 µl fructose. The experimental units were then stored at 5°C.

### Sperm analysis

Sperm concentration was estimated by a spectrophotometry. Sperm motility was assessed as a percentage of progressive sperm motility (forward direction movement). A drop of diluted semen was deposited on a clean glass slide and examined under 400× and then subjectively assessed. Evaluation of spermatozoa motility was done every 24 h until sperm motility decreased to at least 40%. Sperm motility was observed under 100× (for observing the motility of mass spermatozoa) and 400× (for observing the motility of individual spermatozoa). Percent viability was calculated via differential staining using eosin–nigrosin [14]. Slides were used for estimating the percentage of abnormal sperm on the basis of observable abnormalities. A minimum of 200 sperms were counted on each slide for the calculation of live and dead sperms as well as to note the number of abnormal sperms per sample.

### Statistical analysis

Statistical analysis was performed by one-way analysis of variance (ANOVA) with Tukey’s *post hoc* test using SPSS 20.0 software (IBM SPSS, USA).

## Results and Discussion

Good-quality semen required for AI of poultry needs to have more than 90% sperm viability and motility [19]. Data shown in Table-1 indicate that the sample sperm used in our study exhibited values consistent with good-quality sperm.

**Table 1:** Evaluation of fresh rooster spermatozoa (n=10).

Characteristics	Average±SD
Volume (ml)	0.86±0.01
Color	Specific
Consistency	Viscous
Smell	Specific
pH	7.3±0.00
Motility of mass	+++ (3)±0.00
Motility of sperm (%)	95±0.00
Concentration (10^9^ sel/ml)	5.25±0.22
Viability (%)	93±0.5
Sperm abnormalities (%)	6.5±0.01

SD=Standard deviation

### Viability of spermatozoa

The results of post-treatment viability calculations (Table-2) showed significant differences between each treatment (p<0.05). The viability of spermatozoa on day 7 was 46.100% under T2 treatment. This treatment also resulted in the highest percentage of spermatozoa viability compared with other treatments. Qomariyah *et al.*, Getachew *et al.*, and Ihsan also reported similar results [20-22]. Qomariyah [20] showed that a diluent containing 25-30% egg yolk and 70-75% coconut water is the best for maintaining the survival of garut sheep spermatozoa when semen samples were stored at 5°C. Compared to glucose, fructose is a better energy source and can maintain the highest sperm motility [21]; further, a diluent containing 2-30% duck egg yolk is optimal for maintaining Boer goat sperm [22].

**Table 2:** Means of spermatozoa viability with various diluents.

Observation time (h)	Viability (%) (Mean±SD)			

	T0 (Control)	T1	T2	T3
24	0.0610^a^±0.0137	89.3000^c^±0.4831	91.430^d^±0.7304	86.000^b^±0.4714
48	0.0450^a^±0.0053	84.4000^b^±0.6992	87.800^c^±0.7888	84.000^b^±0.6667
72	0.0450^a^±0.0052	75.3000^b^±0.6749	79.400^d^±0.6992	77.9000^c^±0.5676
96	0.0450^a^±0.0053	65.5000^c^±1.7795	68.200 ^d^±0.6325	62.500^b^±0.5271
120	0.0000^a^±0.0000	57.1000^b^±1.1005	59.400^b^±0.8433	56.7000^c^±2.5841
144	0.0000^a^±0.0000	48.1000^b^±0.5677	53.800^c^±0.7888	47.000^b^±4.0552
168	0.0000^a^±0.0000	39.5000^b^±0.5271	46.100^d^±0.5677	40.600^c^±0.9661

Means with different superscripts in a row differ significantly (p≤0.05). SD=Standard deviation

The T2 diluent used in our study contained coconut water, fructose, and egg yolk. Coconut water acts as a good isotonic buffer and supplies nutrients for spermatozoa. Egg yolk as an extracellular cryoprotectant material serves as an energy source and protects extracellular spermatozoa from cold shock because it contains lipoprotein and lecithin [23]. Fructose reduces the damage velocity of sperm membrane permeability so that spermatozoa can live longer [24].

The lowest viability was expected with the T1; coconut water was unable to protect sperm from the effects of cold shock, had lower osmotic pressure as well as decreased pH, and exhibited a toxic effect on seminal plasma [25].

The percentage of sperm viability decreased because over time, the spermatozoa exhibited loss of motility, disruption of cell metabolism, damage to plasma membrane, and finally a decrease in the overall numbers [26]. The viability percentage is usually slightly higher than the motility percentage because spermatozoa can be alive but remain non-motile [27].

### Motility of spermatozoa

The one-way ANOVA tests on sperm motility (Table-3) shows a significant difference (p<0.05) between treatments. It is clear that the motility was affected by the concentrations of the diluent ingredients. Tukey’s *post hoc* test showed that the highest motility was after T2 treatment. On day 7, spermatozoa subjected to T2 treatment showed the highest motility percentage (mean 41.700%), whereas spermatozoa subjected to T1 treatment showed the lowest motility percentage (38.400%). Results observed with the T2 treatment were significantly different from those observed with the other treatments (p<0.05). However, the results observed with the T1 treatment were not significantly different from those of the T3 treatment (motility percentage of 39.700%; p<0.05).

**Table 3:** Means of local rooster spermatozoa motility with various diluents.

Group of treatments	Motility (%) day to						

	1	2	3	4	5	6	7
T0	0.046^a^±0.0699	0.000^a^±0.000	0.000^a^±0.000	0.000^a^±0.000	0.000^a^±0.000	0.000^a^±0.000	0.000^a^±0.000
T1	85.000^c^±0.4714	82.100^a^±0.3162	71.800^b^±0.7888	62.400^c^±0.5164	54.000^c^±0.6667	46.900^b^±1.9120	38.400^b^±1.0750
T2	90.550^d^±1.0124	86.400^d^±0.6992	77.300^c^±0.6749	68.500^d^±0.5270	57.300^d^±1.1595	50.500^c^±2.1213	41.700^c^±1.2517
T3	82.850^b^±0.5798	78.000^b^±1.4907	71.300^b^±0.9487	57.800^b^±0.6325	51.200^b^±1.3166	45.700^b^±1.2517	39.700^b^±1.4181

Means with different superscripts in a row differ significantly (p≤0.05)

High motility was observed at the beginning of the study due to the availability of required energy sources (Table-3). This shows that motility was closely related to spermatozoa metabolism. Adenosine triphosphate (ATP) is a by-product of metabolism and is necessary for spermatozoa motility. When the supply of organic phosphate within ATP and adenosine diphosphate (ADP) is exhausted, the spermatozoa fibril contraction is halted, which leads to the termination of motility. For resuming motility, ATP and ADP need to be replenished via energy from carbohydrates and fats contained in egg yolks [28].

As the energy source depleted over time, a decrease in motility was observed. The abundance of lactic acid that arose due to anaerobic fructose metabolism proved to be toxic to spermatozoa, thus further reducing mobility. In this study, a decrease spermatozoa motility to up to 40% took 7 days after T2 treatment, 6 days after T1 and T3, and <24 h after T0 (control). This indicates that rooster spermatozoa can be effectively stored for up to 7 days at 5°C in a diluent containing 0.274 ml coconut water, 0.12 ml egg yolk, and 6 µl fructose.

### Spermatozoa abnormalities

Results with the T2 diluent showed a significant difference (p<0.05) compared to those of other treatments in terms of abnormalities of spermatozoa morphology (Table-4), including a misshapen head, crooked, shape, or a double tail. Differences in abnormality values between T2 and T3 were not significant, but the lowest abnormality values for sperm were after T2 treatment (6.6800±1.702%). A good diluent provides differential osmotic pressure cryoprotection as well as protection from cold shock [29]. Lecithin from egg yolk can protect against cold shock and provide extracellular cryoprotection. Phospholipids, cholesterol, and low-density lipoproteins within egg yolk may also be factors that provide protection against cold shock [30]. Coconut water is a natural isotonic liquid that reduces morphological membrane damage, and fructose serves as a specific organic compound for spermatozoa.

**Table 4:** Means of percentages of spermatozoa abnormalities.

Group of treatments	Average±SD
T0	19.2070c±3.553
T1	10.5720b±1.098
T2	6.6800a±1.702
T3	7.7800a±1.002

Means with different superscripts in a row differ significantly (p≤0.05). SD=Standard deviation

## Conclusion

A diluent containing a combination of coconut water, egg yolk, and fructose can be added to rooster sperm to increase spermatozoa motility and viability for up to 7 days when the semen samples are stored at 5°C. However, further studies are needed to understand the mechanisms of action and the actual impact on fertility outputs.

## Author’s Contributions

SER and MSS carried out the main research works, MSS performed the analysis of data, and SER prepared the manuscript and revised the manuscript. All of the authors have read and approved the final manuscript.
